# Tetra-μ-benzoato-bis­[(3-methyl­quinoline)copper(II)](*Cu—Cu*)

**DOI:** 10.1107/S1600536808024859

**Published:** 2008-08-09

**Authors:** Byeong Kwon Park, Kyung-Hwan Jang, Pan-Gi Kim, Cheal Kim, Youngmee Kim

**Affiliations:** a Department of Fine Chemistry, and Eco-Products and Materials Education Center, Seoul National University of Technology, Seoul 139-743, Republic of Korea; b Forest Practice Research Center, Pocheon-Si, Gyeonggi-Do 487-821, Republic of Korea; c Department of Forest and Environmental Resources, Kyungpook National University, Sangju, 742-711, Republic of Korea; d Department of Chemistry and Nano Science, Ewha Womans University, Seoul 120-750, Republic of Korea

## Abstract

In the title compound, [Cu_2_(C_7_H_5_O_2_)_4_(C_10_H_9_N)_2_], the paddle-wheel-type dinuclear complex mol­ecule contains four bridging benzoate groups and two terminal 3-methyl­quinoline ligands. The asymmetric unit contains one and a half mol­ecules with a total of three independent Cu atoms; there is an inversion center at the mid-point of the Cu⋯Cu bond in one molecule. The octa­hedral coordination of each Cu atom, with four O atoms in the equatorial plane, is completed by an N atom of a 3-methyl­quinoline ligand [Cu—N = 2.190 (4)–2.203 (3) Å] and by another Cu atom [Cu⋯Cu = 2.667 (1) and 2.6703 (7) Å]. The Cu atoms are all *ca* 0.22 Å out of the plane of the four bonded O atoms.

## Related literature

For related literature, see: Daniele *et al.* (2008 ▶); Lee *et al.* (2008 ▶); Parkin (2004 ▶); Tshuva & Lippard (2004 ▶); Wu *et al.* (2004 ▶); Weston (2005 ▶); Yu *et al.* (2008 ▶).
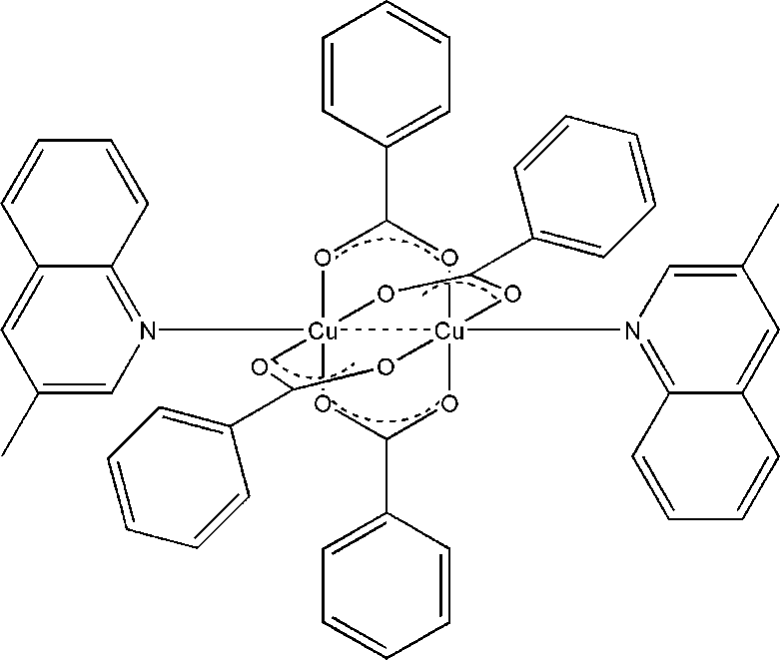


## Experimental

### 

#### Crystal data

[Cu_2_(C_7_H_5_O_2_)_4_(C_10_H_9_N)_2_]
*M* *_r_* = 897.88
Monoclinic, 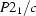

*a* = 29.988 (4) Å
*b* = 16.6892 (19) Å
*c* = 12.5972 (15) Å
β = 90.45°
*V* = 6304.4 (13) Å^3^
*Z* = 6
Mo *K*α radiation
μ = 1.07 mm^−1^
*T* = 293 (2) K
0.08 × 0.08 × 0.05 mm

#### Data collection

Bruker SMART CCD diffractometer
Absorption correction: multi-scan (*SADABS*; Bruker, 1997 ▶) *T*_min_ = 0.918, *T*_max_ = 0.948
35088 measured reflections
12366 independent reflections
6309 reflections with *I* > 2σ(*I*)
*R*_int_ = 0.080

#### Refinement

*R*[*F* ^2^ > 2σ(*F* ^2^)] = 0.054
*wR*(*F* ^2^) = 0.125
*S* = 0.97
12366 reflections
814 parameters
H-atom parameters constrained
Δρ_max_ = 0.34 e Å^−3^
Δρ_min_ = −0.38 e Å^−3^

### 

Data collection: *SMART* (Bruker, 1997 ▶); cell refinement: *SAINT* (Bruker, 1997 ▶); data reduction: *SAINT*; program(s) used to solve structure: *SHELXS97* (Sheldrick, 2008 ▶); program(s) used to refine structure: *SHELXL97* (Sheldrick, 2008 ▶); molecular graphics: *SHELXTL* (Sheldrick, 2008 ▶); software used to prepare material for publication: *SHELXTL*.

## Supplementary Material

Crystal structure: contains datablocks I, global. DOI: 10.1107/S1600536808024859/lh2672sup1.cif

Structure factors: contains datablocks I. DOI: 10.1107/S1600536808024859/lh2672Isup2.hkl

Additional supplementary materials:  crystallographic information; 3D view; checkCIF report

## Figures and Tables

**Fig. 1.**
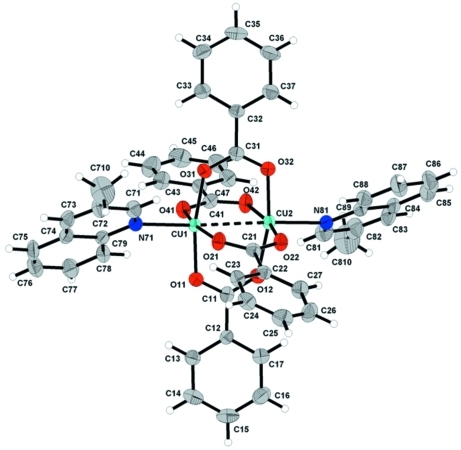
The molecular structure of one independent molecule of the title compound showing the atom-labeling scheme. Displacement ellipsoids are shown at the 30% probability level.

**Fig. 2.**
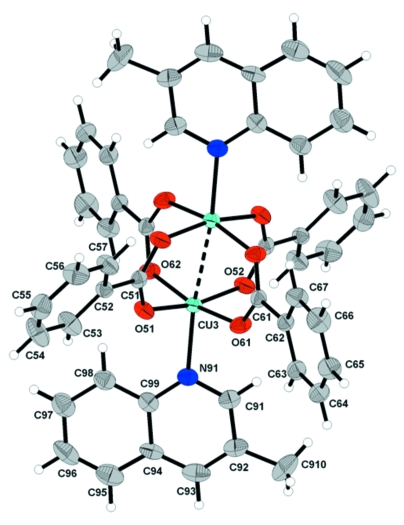
The molecular structure of the other indpendent moleclue of the title compound showing the atom-labeling scheme. Displacement ellipsoids are shown at the 30% probability level. Unlabeled atoms are related by the symmetry operator (-x + 1,-y + 1,-z + 1).

## supplementary crystallographic information

### Comment

Transitionmetal ions as well as sodium, potassium, calcium, and magnesium ions are the major cation contributors to the inorganic composition of natural water and biological fluids (Daniele, *et al.*, 2008). Therefore, coordination chemistry of transition metal ions in many environmental and biological processes such as membranes transport or metal metabolism was well investigated (Parkin, 2004; Tshuva & Lippard 2004; Weston, 2005; Wu *et al.*,  2004), especially if compared to alkali metal ions. While the main interest is focused on the interaction of transition metal ions with biologically active molecules such as amino acids, proteins, sugars, nucleotides *etc*, the interaction of the transition metal ions with fulvic acids and humic acids, which are mainly found in soil, is less studied. As models to examine the interaction, therefore, we have previously used copper(II) benzoate as a building block and reported the structures of copper(II) benzoates with quinoxaline and 6-methylquinoline (Lee, *et al.*, 2008; Yu, *et al.*, 2008). In this work, we have employed 3-methylquinoline to further investigate the substituent effects of organic ligands on the structure of copper-benzoate containing coordination complexes. We report herein the crystal structure of the product of the reaction of copper(II)benzoate with 3-methylquinoline.

The asymmetric unit contains one and half molecules containing three independent Cu atoms, and there is an inversion center at the mid-point of the Cu3···Cu3 bond [symmetry operation (-*x* + 1,-*y* + 1,-*z* + 1)]. The complex has a paddle-wheel type dinuclear copper-benzoate conformation (Figs. 1 & 2). This constructed by four bridging benzoate groups and two terminal 3-methylquinoline ligands. The octahedral coordination of each Cu atom, with four oxygen atoms in the equatorial plane, is completed by a nitrogen atom of 3-methylquinoline molecule (Cu—N 2.190 (4) – 2.203 (3) Å) and by anothercopper atom (Cu···Cu 2.667 (1) and 2.6703 (7) Å). The copper atoms are all ca. 0.22 Å out of the plane of the four oxygen atoms.

### Experimental

19.0 mg (0.1 mmol) of Cu(NO_3_)_2_^.^2.5H_2_O and 28.0 mg (0.2 mmol) of C_6_H_5_COONH_4 _were dissolved in 4 ml methanol and carefully layered by 4 ml methylene chloride solution of 3-methylquinoline ligand (29.0 mg, 0.2 mmol). Suitable crystals of the title compound were obtained in a few weeks.

### Refinement

H atoms were placed in calculated positions with C-H distances of 0.93 Å (benzene) and 0.96 Å  (methyl). They were included in the refinement in riding-motion approximation with U_iso_(H) = 1.2U_eq_(C) or 2.5U_eq_(C) for methyl.

### Crystal data

**Table:**

[Cu_2_(C_7_H_5_O_2_)_4_(C_10_H_9_N)_2_]	*F*_000_ = 2772
*M**_r_* = 897.88	*D*_x_ = 1.419 Mg m^−^^3^
Monoclinic, *P*2_1_/*c*	Mo *K*α radiation λ = 0.71073 Å
Hall symbol: -P 2ybc	Cell parameters from 2186 reflections
*a* = 29.988 (4) Å	θ = 2.4–19.7º
*b* = 16.6892 (19) Å	µ = 1.07 mm^−^^1^
*c* = 12.5972 (15) Å	*T* = 293 (2) K
β = 90.45º	Block, blue
*V* = 6304.4 (13) Å^3^	0.08 × 0.08 × 0.05 mm
*Z* = 6	

### Data collection

**Table:**

Bruker SMART-CCD diffractometer	12366 independent reflections
Radiation source: fine-focus sealed tube	6309 reflections with *I* > 2σ(*I*)
Monochromator: graphite	*R*_int_ = 0.080
*T* = 293(2) K	θ_max_ = 26.0º
φ and ω scans	θ_min_ = 1.8º
Absorption correction: multi-scan(SADABS; Bruker, 1997)	*h* = −25→36
*T*_min_ = 0.918, *T*_max_ = 0.948	*k* = −16→20
35088 measured reflections	*l* = −14→15

### Refinement

**Table:**

Refinement on *F*^2^	Secondary atom site location: difference Fourier map
Least-squares matrix: full	Hydrogen site location: inferred from neighbouring sites
*R*[*F*^2^ > 2σ(*F*^2^)] = 0.054	H-atom parameters constrained
*wR*(*F*^2^) = 0.125	*w* = 1/[σ^2^(*F*_o_^2^) + (0.0215*P*)^2^] where *P* = (*F*_o_^2^ + 2*F*_c_^2^)/3
*S* = 0.97	(Δ/σ)_max_ = 0.001
12366 reflections	Δρ_max_ = 0.34 e Å^−^^3^
814 parameters	Δρ_min_ = −0.38 e Å^−^^3^
Primary atom site location: structure-invariant direct methods	Extinction correction: none

### Special details

**Table:**

Geometry. All e.s.d.'s (except the e.s.d. in the dihedral angle between two l.s. planes) are estimated using the full covariance matrix. The cell e.s.d.'s are taken into account individually in the estimation of e.s.d.'s in distances, angles and torsion angles; correlations between e.s.d.'s in cell parameters are only used when they are defined by crystal symmetry. An approximate (isotropic) treatment of cell e.s.d.'s is used for estimating e.s.d.'s involving l.s. planes.
Refinement. Refinement of *F*^2^ against ALL reflections. The weighted *R*-factor *wR* and goodness of fit *S* are based on *F*^2^, conventional *R*-factors *R* are based on *F*, with *F* set to zero for negative *F*^2^. The threshold expression of *F*^2^ > σ(*F*^2^) is used only for calculating *R*-factors(gt) *etc*. and is not relevant to the choice of reflections for refinement. *R*-factors based on *F*^2^ are statistically about twice as large as those based on *F*, and *R*- factors based on ALL data will be even larger.

### Fractional atomic coordinates and isotropic or equivalent isotropic displacement parameters (Å^2^)

**Table:**

	*x*	*y*	*z*	*U*_iso_*/*U*_eq_	
Cu1	0.140873 (17)	0.48955 (3)	1.08169 (4)	0.03683 (15)	
Cu2	0.195328 (17)	0.51147 (3)	0.91752 (4)	0.03964 (15)	
Cu3	0.472853 (17)	0.48903 (3)	0.41692 (4)	0.03757 (15)	
O11	0.10706 (10)	0.41783 (15)	0.9886 (2)	0.0492 (8)	
O12	0.15067 (11)	0.44098 (17)	0.8491 (2)	0.0557 (9)	
C11	0.11946 (15)	0.4046 (3)	0.8941 (4)	0.0422 (11)	
C12	0.09454 (15)	0.3426 (2)	0.8329 (3)	0.0402 (11)	
C13	0.05386 (16)	0.3141 (3)	0.8657 (3)	0.0529 (13)	
H13	0.0416	0.3328	0.9286	0.063*	
C14	0.03133 (19)	0.2582 (3)	0.8059 (4)	0.0678 (15)	
H14	0.0033	0.2407	0.8271	0.081*	
C15	0.0498 (2)	0.2278 (3)	0.7153 (4)	0.0686 (16)	
H15	0.0345	0.1896	0.6752	0.082*	
C16	0.0906 (2)	0.2542 (3)	0.6844 (4)	0.0636 (16)	
H16	0.1037	0.2328	0.6239	0.088*	
C17	0.11279 (17)	0.3123 (3)	0.7420 (4)	0.0609 (14)	
H17	0.1403	0.3311	0.7190	0.073*	
O21	0.18305 (10)	0.39938 (16)	1.1068 (2)	0.0500 (8)	
O22	0.22602 (10)	0.41433 (16)	0.9634 (2)	0.0518 (8)	
C21	0.21187 (15)	0.3754 (2)	1.0430 (4)	0.0430 (11)	
C22	0.23111 (14)	0.2939 (2)	1.0597 (3)	0.0430 (11)	
C23	0.22233 (15)	0.2506 (3)	1.1509 (4)	0.0498 (12)	
H23	0.2056	0.2738	1.2047	0.060*	
C24	0.23802 (17)	0.1737 (3)	1.1631 (4)	0.0602 (14)	
H24	0.2320	0.1454	1.2249	0.072*	
C25	0.26228 (18)	0.1389 (3)	1.0851 (5)	0.0682 (15)	
H25	0.2726	0.0868	1.0936	0.082*	
C26	0.27169 (18)	0.1805 (3)	0.9935 (4)	0.0632 (16)	
H26	0.2883	0.1567	0.9401	0.088*	
C27	0.25611 (17)	0.2581 (3)	0.9816 (4)	0.0599 (14)	
H27	0.2626	0.2864	0.9200	0.072*	
O31	0.18253 (10)	0.56428 (17)	1.1485 (2)	0.0508 (8)	
O32	0.23127 (10)	0.57679 (16)	1.0156 (2)	0.0530 (8)	
C31	0.21740 (16)	0.5932 (2)	1.1076 (4)	0.0434 (11)	
C32	0.24312 (16)	0.6521 (2)	1.1726 (3)	0.0437 (11)	
C33	0.22318 (17)	0.6889 (3)	1.2578 (4)	0.0573 (13)	
H33	0.1938	0.6767	1.2747	0.069*	
C34	0.2467 (2)	0.7440 (3)	1.3184 (4)	0.0667 (15)	
H34	0.2328	0.7703	1.3742	0.080*	
C35	0.2909 (2)	0.7598 (3)	1.2959 (4)	0.0614 (16)	
H35	0.3070	0.7951	1.3384	0.086*	
C36	0.31071 (19)	0.7237 (3)	1.2116 (5)	0.0641 (18)	
H36	0.3403	0.7349	1.1960	0.101*	
C37	0.28705 (18)	0.6705 (3)	1.1492 (4)	0.0683 (15)	
H37	0.3006	0.6468	1.0910	0.082*	
O41	0.10742 (10)	0.58178 (15)	1.0264 (2)	0.0494 (8)	
O42	0.15600 (11)	0.60454 (16)	0.8965 (2)	0.0542 (8)	
C41	0.12160 (16)	0.6212 (2)	0.9483 (3)	0.0429 (11)	
C42	0.09650 (15)	0.6947 (2)	0.9168 (3)	0.0431 (11)	
C43	0.06066 (17)	0.7200 (3)	0.9734 (4)	0.0592 (14)	
H43	0.0518	0.6910	1.0326	0.071*	
C44	0.03721 (19)	0.7880 (3)	0.9447 (4)	0.0686 (17)	
H44	0.0125	0.8041	0.9833	0.094*	
C45	0.0510 (2)	0.8319 (3)	0.8577 (5)	0.0684 (18)	
H45	0.0358	0.8782	0.8383	0.094*	
C46	0.08665 (19)	0.8073 (3)	0.8008 (4)	0.0665 (15)	
H46	0.0959	0.8370	0.7425	0.080*	
C47	0.10937 (17)	0.7380 (3)	0.8291 (4)	0.0575 (13)	
H47	0.1334	0.7207	0.7888	0.069*	
O51	0.43770 (10)	0.42328 (16)	0.5140 (2)	0.0498 (8)	
O52	0.51619 (11)	0.55942 (17)	0.3479 (2)	0.0530 (8)	
C51	0.45058 (15)	0.4085 (2)	0.6069 (4)	0.0395 (11)	
C52	0.42434 (15)	0.3491 (2)	0.6706 (3)	0.0409 (11)	
C53	0.38141 (18)	0.3293 (3)	0.6424 (4)	0.0675 (15)	
H53	0.3683	0.3524	0.5826	0.081*	
C54	0.35739 (19)	0.2746 (3)	0.7030 (5)	0.0642 (18)	
H54	0.3282	0.2617	0.6844	0.101*	
C55	0.3771 (2)	0.2404 (3)	0.7899 (5)	0.0635 (17)	
H55	0.3609	0.2048	0.8315	0.088*	
C56	0.4198 (2)	0.2578 (3)	0.8159 (4)	0.0646 (17)	
H56	0.4335	0.2324	0.8734	0.089*	
C57	0.44345 (17)	0.3137 (3)	0.7565 (4)	0.0621 (14)	
H57	0.4725	0.3269	0.7760	0.075*	
O61	0.51297 (11)	0.39581 (16)	0.3964 (2)	0.0530 (8)	
O62	0.44192 (10)	0.58609 (16)	0.4637 (2)	0.0515 (8)	
C61	0.54343 (15)	0.3740 (2)	0.4592 (4)	0.0415 (11)	
C62	0.56345 (14)	0.2932 (2)	0.4413 (4)	0.0432 (11)	
C63	0.55385 (16)	0.2511 (3)	0.3497 (4)	0.0548 (13)	
H63	0.5362	0.2744	0.2970	0.066*	
C64	0.57037 (17)	0.1745 (3)	0.3357 (4)	0.0638 (14)	
H64	0.5644	0.1467	0.2732	0.077*	
C65	0.59534 (19)	0.1400 (3)	0.4133 (5)	0.0642 (17)	
H65	0.6060	0.0881	0.4043	0.089*	
C66	0.60512 (18)	0.1812 (3)	0.5057 (5)	0.0640 (16)	
H66	0.6222	0.1572	0.5587	0.089*	
C67	0.58936 (16)	0.2582 (3)	0.5191 (4)	0.0581 (13)	
H67	0.5963	0.2865	0.5806	0.070*	
N71	0.10380 (13)	0.46908 (19)	1.2285 (3)	0.0431 (9)	
C71	0.12994 (17)	0.4504 (3)	1.3107 (3)	0.0545 (13)	
H71	0.1606	0.4505	1.3004	0.065*	
C72	0.1144 (2)	0.4307 (3)	1.4107 (4)	0.0668 (15)	
C73	0.0702 (2)	0.4327 (3)	1.4260 (4)	0.0616 (14)	
H73	0.0592	0.4214	1.4932	0.074*	
C74	0.03990 (18)	0.4512 (2)	1.3439 (4)	0.0508 (12)	
C75	−0.0065 (2)	0.4526 (3)	1.3547 (5)	0.0602 (16)	
H75	−0.0191	0.4424	1.4205	0.084*	
C76	−0.0333 (2)	0.4688 (3)	1.2691 (5)	0.0686 (17)	
H76	−0.0641	0.4682	1.2766	0.094*	
C77	−0.01478 (18)	0.4861 (3)	1.1709 (4)	0.0660 (14)	
H77	−0.0332	0.4974	1.1131	0.079*	
C78	0.03068 (16)	0.4866 (2)	1.1591 (4)	0.0518 (12)	
H78	0.0428	0.4989	1.0933	0.062*	
C79	0.05883 (16)	0.4691 (2)	1.2434 (3)	0.0422 (11)	
C710	0.1480 (2)	0.4085 (4)	1.4960 (4)	0.0720 (3)	
H71A	0.1378	0.4275	1.5635	0.180*	
H71B	0.1763	0.4326	1.4802	0.180*	
H71C	0.1513	0.3513	1.4985	0.180*	
N81	0.22867 (14)	0.5391 (2)	0.7666 (3)	0.0496 (10)	
C81	0.2004 (2)	0.5688 (3)	0.6934 (4)	0.0684 (16)	
H81	0.1701	0.5656	0.7076	0.082*	
C82	0.2129 (2)	0.6040 (3)	0.5976 (4)	0.0613 (18)	
C83	0.2566 (2)	0.6056 (3)	0.5763 (4)	0.0699 (18)	
H83	0.2661	0.6280	0.5128	0.096*	
C84	0.2888 (2)	0.5740 (3)	0.6477 (4)	0.0622 (15)	
C85	0.3341 (3)	0.5753 (3)	0.6302 (6)	0.063 (2)	
H85	0.3450	0.5970	0.5676	0.111*	
C86	0.3636 (2)	0.5446 (4)	0.7051 (7)	0.0701 (2)	
H86	0.3941	0.5460	0.6922	0.121*	
C87	0.3484 (2)	0.5122 (3)	0.7975 (5)	0.0686 (19)	
H87	0.3683	0.4909	0.8469	0.106*	
C88	0.3032 (2)	0.5113 (3)	0.8172 (5)	0.0672 (15)	
H88	0.2931	0.4897	0.8807	0.081*	
C89	0.27255 (19)	0.5416 (3)	0.7454 (4)	0.0560 (13)	
C810	0.1772 (2)	0.6378 (4)	0.5262 (5)	0.0731 (3)	
H81A	0.1760	0.6950	0.5344	0.196*	
H81B	0.1489	0.6152	0.5450	0.196*	
H81C	0.1837	0.6249	0.4538	0.196*	
N91	0.43539 (14)	0.46388 (19)	0.2708 (3)	0.0485 (10)	
C91	0.46191 (19)	0.4377 (3)	0.1929 (4)	0.0633 (14)	
H91	0.4925	0.4395	0.2047	0.076*	
C92	0.4469 (2)	0.4079 (3)	0.0954 (4)	0.0673 (17)	
C93	0.4021 (2)	0.4055 (3)	0.0797 (4)	0.0669 (17)	
H93	0.3908	0.3853	0.0162	0.092*	
C94	0.37281 (19)	0.4328 (3)	0.1566 (4)	0.0547 (13)	
C95	0.3267 (2)	0.4328 (3)	0.1451 (5)	0.0678 (17)	
H95	0.3142	0.4134	0.0823	0.093*	
C96	0.2988 (2)	0.4602 (3)	0.2227 (6)	0.0642 (19)	
H96	0.2681	0.4600	0.2119	0.101*	
C97	0.3167 (2)	0.4883 (3)	0.3172 (5)	0.0675 (17)	
H97	0.2980	0.5066	0.3705	0.093*	
C98	0.36280 (17)	0.4889 (3)	0.3322 (4)	0.0584 (13)	
H98	0.3748	0.5077	0.3958	0.070*	
C99	0.39083 (17)	0.4620 (2)	0.2538 (4)	0.0482 (12)	
C910	0.4806 (2)	0.3794 (5)	0.0176 (5)	0.0649 (3)	
H91A	0.4849	0.3227	0.0258	0.224*	
H91B	0.5084	0.4066	0.0300	0.224*	
H91C	0.4703	0.3906	−0.0532	0.224*	

### Atomic displacement parameters (Å^2^)

**Table:**

	*U*^11^	*U*^22^	*U*^33^	*U*^12^	*U*^13^	*U*^23^
Cu1	0.0371 (3)	0.0385 (3)	0.0350 (3)	0.0012 (2)	0.0050 (2)	0.0032 (2)
Cu2	0.0409 (3)	0.0401 (3)	0.0380 (3)	0.0014 (3)	0.0081 (2)	0.0051 (2)
Cu3	0.0374 (3)	0.0370 (3)	0.0382 (3)	0.0004 (2)	−0.0055 (2)	−0.0039 (2)
O11	0.050 (2)	0.0472 (18)	0.051 (2)	−0.0056 (15)	0.0068 (16)	−0.0048 (15)
O12	0.063 (2)	0.063 (2)	0.0413 (18)	−0.0214 (18)	0.0092 (17)	−0.0030 (15)
C11	0.036 (3)	0.049 (3)	0.042 (3)	0.011 (2)	−0.004 (2)	0.005 (2)
C12	0.048 (3)	0.038 (3)	0.035 (2)	0.003 (2)	0.002 (2)	0.002 (2)
C13	0.055 (4)	0.055 (3)	0.049 (3)	−0.007 (3)	0.001 (3)	−0.001 (2)
C14	0.069 (4)	0.057 (3)	0.077 (4)	−0.018 (3)	−0.010 (3)	0.004 (3)
C15	0.092 (5)	0.043 (3)	0.070 (4)	−0.008 (3)	−0.026 (3)	0.000 (3)
C16	0.088 (5)	0.068 (4)	0.064 (4)	0.001 (3)	0.007 (3)	−0.016 (3)
C17	0.061 (4)	0.059 (3)	0.063 (3)	−0.006 (3)	0.003 (3)	−0.010 (3)
O21	0.053 (2)	0.0483 (18)	0.0493 (19)	0.0148 (16)	0.0117 (16)	0.0093 (15)
O22	0.056 (2)	0.0444 (18)	0.055 (2)	0.0084 (15)	0.0154 (17)	0.0118 (15)
C21	0.037 (3)	0.043 (3)	0.049 (3)	0.001 (2)	−0.007 (2)	0.000 (2)
C22	0.035 (3)	0.043 (3)	0.051 (3)	0.000 (2)	−0.006 (2)	−0.001 (2)
C23	0.045 (3)	0.048 (3)	0.056 (3)	0.003 (2)	0.003 (2)	0.009 (2)
C24	0.058 (4)	0.049 (3)	0.074 (4)	0.002 (3)	−0.003 (3)	0.019 (3)
C25	0.069 (4)	0.040 (3)	0.095 (4)	0.012 (3)	−0.012 (3)	0.008 (3)
C26	0.079 (4)	0.056 (3)	0.086 (4)	0.018 (3)	0.023 (3)	−0.006 (3)
C27	0.068 (4)	0.044 (3)	0.068 (3)	0.010 (3)	0.009 (3)	0.008 (3)
O31	0.047 (2)	0.0543 (19)	0.0508 (19)	−0.0127 (16)	0.0085 (16)	−0.0036 (15)
O32	0.053 (2)	0.0517 (19)	0.054 (2)	−0.0087 (15)	0.0080 (17)	−0.0091 (16)
C31	0.046 (3)	0.037 (3)	0.047 (3)	0.001 (2)	−0.005 (2)	0.004 (2)
C32	0.047 (3)	0.030 (2)	0.054 (3)	0.001 (2)	−0.004 (2)	0.002 (2)
C33	0.061 (4)	0.059 (3)	0.052 (3)	−0.008 (3)	0.000 (3)	−0.005 (3)
C34	0.091 (5)	0.060 (3)	0.050 (3)	−0.010 (3)	−0.002 (3)	−0.011 (3)
C35	0.080 (5)	0.045 (3)	0.089 (4)	−0.014 (3)	−0.028 (4)	−0.007 (3)
C36	0.059 (4)	0.059 (4)	0.095 (6)	−0.018 (3)	0.002 (4)	−0.025 (4)
C37	0.054 (4)	0.052 (3)	0.080 (4)	−0.007 (3)	0.015 (3)	−0.017 (3)
O41	0.050 (2)	0.0431 (18)	0.055 (2)	0.0095 (15)	0.0088 (16)	0.0144 (15)
O42	0.057 (2)	0.0524 (19)	0.053 (2)	0.0165 (17)	0.0169 (17)	0.0152 (15)
C41	0.051 (3)	0.039 (3)	0.039 (3)	0.008 (2)	−0.004 (2)	0.001 (2)
C42	0.044 (3)	0.036 (3)	0.049 (3)	0.002 (2)	−0.001 (2)	0.004 (2)
C43	0.066 (4)	0.048 (3)	0.063 (3)	0.014 (3)	0.004 (3)	0.007 (3)
C44	0.088 (5)	0.059 (4)	0.089 (4)	0.028 (3)	0.009 (4)	0.001 (3)
C45	0.083 (5)	0.046 (3)	0.086 (5)	0.012 (3)	−0.018 (4)	0.016 (3)
C46	0.073 (4)	0.049 (3)	0.078 (4)	0.000 (3)	−0.003 (3)	0.024 (3)
C47	0.058 (4)	0.048 (3)	0.066 (3)	0.003 (3)	0.000 (3)	0.014 (3)
O51	0.043 (2)	0.0537 (19)	0.052 (2)	−0.0098 (15)	−0.0096 (16)	0.0077 (16)
O52	0.053 (2)	0.0570 (19)	0.0490 (19)	−0.0170 (17)	−0.0084 (16)	−0.0001 (15)
C51	0.040 (3)	0.030 (2)	0.049 (3)	0.007 (2)	0.004 (2)	−0.003 (2)
C52	0.040 (3)	0.038 (3)	0.044 (3)	−0.002 (2)	0.000 (2)	−0.002 (2)
C53	0.056 (4)	0.052 (3)	0.094 (4)	−0.007 (3)	−0.014 (3)	0.021 (3)
C54	0.051 (4)	0.058 (4)	0.093 (6)	−0.016 (3)	−0.001 (4)	0.028 (4)
C55	0.082 (5)	0.053 (3)	0.085 (4)	−0.008 (3)	0.027 (4)	0.006 (3)
C56	0.092 (5)	0.071 (4)	0.060 (4)	−0.015 (3)	−0.012 (3)	0.013 (3)
C57	0.056 (4)	0.072 (3)	0.058 (3)	−0.019 (3)	−0.007 (3)	0.011 (3)
O61	0.060 (2)	0.0472 (19)	0.052 (2)	0.0174 (17)	−0.0142 (17)	−0.0129 (15)
O62	0.054 (2)	0.0437 (18)	0.056 (2)	0.0123 (15)	−0.0148 (17)	−0.0144 (15)
C61	0.038 (3)	0.038 (3)	0.049 (3)	0.002 (2)	0.006 (2)	−0.004 (2)
C62	0.041 (3)	0.034 (3)	0.055 (3)	0.004 (2)	0.004 (2)	−0.009 (2)
C63	0.052 (3)	0.047 (3)	0.066 (3)	0.001 (2)	0.005 (3)	−0.008 (3)
C64	0.062 (4)	0.055 (3)	0.075 (4)	0.005 (3)	0.005 (3)	−0.021 (3)
C65	0.072 (4)	0.037 (3)	0.084 (5)	0.001 (3)	0.004 (4)	−0.020 (3)
C66	0.070 (4)	0.051 (3)	0.080 (4)	0.015 (3)	−0.008 (3)	0.009 (3)
C67	0.061 (4)	0.040 (3)	0.073 (4)	0.005 (2)	−0.009 (3)	−0.004 (2)
N71	0.050 (3)	0.042 (2)	0.038 (2)	−0.0008 (18)	0.0053 (19)	0.0046 (17)
C71	0.059 (4)	0.062 (3)	0.043 (3)	−0.002 (3)	0.001 (3)	0.012 (2)
C72	0.066 (4)	0.087 (4)	0.047 (3)	0.004 (3)	0.000 (3)	0.015 (3)
C73	0.083 (5)	0.064 (3)	0.038 (3)	0.004 (3)	0.018 (3)	0.007 (2)
C74	0.065 (4)	0.038 (3)	0.050 (3)	−0.001 (2)	0.017 (3)	0.002 (2)
C75	0.068 (4)	0.072 (4)	0.071 (4)	0.000 (3)	0.039 (3)	0.006 (3)
C76	0.049 (4)	0.084 (4)	0.083 (5)	0.000 (3)	0.021 (4)	0.006 (4)
C77	0.052 (4)	0.070 (3)	0.076 (4)	0.007 (3)	0.002 (3)	0.008 (3)
C78	0.049 (3)	0.054 (3)	0.052 (3)	0.006 (3)	0.011 (2)	0.008 (2)
C79	0.052 (3)	0.035 (3)	0.040 (3)	−0.002 (2)	0.006 (2)	0.001 (2)
C710	0.108 (6)	0.095 (7)	0.066 (4)	−0.003 (5)	−0.026 (4)	0.046 (4)
N81	0.056 (3)	0.047 (2)	0.046 (2)	−0.008 (2)	0.015 (2)	−0.0016 (19)
C81	0.092 (5)	0.069 (4)	0.044 (3)	−0.006 (3)	0.003 (3)	0.010 (3)
C82	0.099 (6)	0.092 (4)	0.053 (4)	−0.003 (4)	0.013 (4)	0.013 (3)
C83	0.083 (6)	0.083 (4)	0.054 (4)	−0.011 (4)	0.035 (4)	−0.003 (3)
C84	0.072 (4)	0.051 (3)	0.063 (4)	−0.015 (3)	0.032 (3)	−0.012 (3)
C85	0.082 (6)	0.066 (4)	0.111 (6)	−0.009 (4)	0.054 (5)	−0.012 (4)
C86	0.068 (5)	0.071 (4)	0.095 (8)	−0.001 (4)	0.054 (5)	−0.008 (5)
C87	0.071 (5)	0.078 (4)	0.088 (5)	0.003 (3)	0.034 (4)	0.010 (4)
C88	0.072 (4)	0.048 (3)	0.082 (4)	−0.004 (3)	0.023 (3)	0.001 (3)
C89	0.063 (4)	0.041 (3)	0.064 (3)	−0.007 (3)	0.022 (3)	−0.011 (2)
C810	0.082 (7)	0.098 (7)	0.081 (5)	0.020 (6)	−0.020 (5)	0.046 (5)
N91	0.053 (3)	0.042 (2)	0.050 (2)	0.0013 (19)	−0.009 (2)	−0.0012 (18)
C91	0.067 (4)	0.074 (4)	0.049 (3)	−0.003 (3)	−0.003 (3)	−0.009 (3)
C92	0.080 (5)	0.099 (4)	0.053 (4)	−0.004 (4)	−0.011 (3)	−0.021 (3)
C93	0.094 (5)	0.075 (4)	0.061 (4)	−0.009 (4)	−0.030 (4)	−0.003 (3)
C94	0.062 (4)	0.041 (3)	0.061 (3)	−0.010 (3)	−0.022 (3)	0.000 (2)
C95	0.084 (5)	0.063 (4)	0.086 (5)	−0.007 (3)	−0.027 (4)	0.008 (3)
C96	0.053 (4)	0.073 (4)	0.126 (6)	−0.006 (3)	−0.031 (4)	0.007 (4)
C97	0.061 (4)	0.067 (4)	0.104 (5)	0.002 (3)	−0.008 (4)	0.000 (3)
C98	0.059 (4)	0.048 (3)	0.068 (3)	−0.002 (3)	−0.007 (3)	−0.001 (3)
C99	0.050 (3)	0.032 (3)	0.062 (3)	0.000 (2)	−0.005 (3)	0.008 (2)
C910	0.089 (7)	0.097 (9)	0.082 (5)	0.005 (7)	0.031 (5)	−0.054 (5)

### Geometric parameters (Å, °)

**Table:**

Cu1—O31	1.952 (3)	C55—C56	1.354 (7)
Cu1—O11	1.954 (3)	C55—H55	0.9300
Cu1—O41	1.962 (3)	C56—C57	1.393 (6)
Cu1—O21	1.990 (3)	C56—H56	0.9300
Cu1—N71	2.192 (3)	C57—H57	0.9300
Cu1—Cu2	2.6703 (7)	O61—C61	1.257 (5)
Cu2—O22	1.950 (3)	O62—C61^i^	1.255 (5)
Cu2—O32	1.963 (3)	C61—O62^i^	1.255 (5)
Cu2—O42	1.967 (3)	C61—C62	1.494 (5)
Cu2—O12	1.975 (3)	C62—C67	1.375 (6)
Cu2—N81	2.203 (3)	C62—C63	1.380 (6)
Cu3—O51	1.957 (3)	C63—C64	1.384 (6)
Cu3—O62	1.960 (3)	C63—H63	0.9300
Cu3—O52	1.960 (3)	C64—C65	1.355 (7)
Cu3—O61	1.985 (3)	C64—H64	0.9300
Cu3—N91	2.190 (4)	C65—C66	1.383 (7)
Cu3—Cu3^i^	2.6671 (10)	C65—H65	0.9300
O11—C11	1.270 (5)	C66—C67	1.380 (6)
O12—C11	1.254 (5)	C66—H66	0.9300
C11—C12	1.488 (6)	C67—H67	0.9300
C12—C17	1.370 (6)	N71—C71	1.331 (5)
C12—C13	1.376 (6)	N71—C79	1.363 (5)
C13—C14	1.375 (6)	C71—C72	1.385 (6)
C13—H13	0.9300	C71—H71	0.9300
C14—C15	1.369 (7)	C72—C73	1.343 (7)
C14—H14	0.9300	C72—C710	1.513 (7)
C15—C16	1.362 (7)	C73—C74	1.406 (6)
C15—H15	0.9300	C73—H73	0.9300
C16—C17	1.380 (6)	C74—C75	1.399 (7)
C16—H16	0.9300	C74—C79	1.423 (6)
C17—H17	0.9300	C75—C76	1.367 (7)
O21—C21	1.250 (5)	C75—H75	0.9300
O22—C21	1.270 (5)	C76—C77	1.391 (7)
C21—C22	1.492 (5)	C76—H76	0.9300
C22—C27	1.378 (6)	C77—C78	1.373 (6)
C22—C23	1.385 (5)	C77—H77	0.9300
C23—C24	1.374 (6)	C78—C79	1.382 (6)
C23—H23	0.9300	C78—H78	0.9300
C24—C25	1.357 (6)	C710—H71A	0.9600
C24—H24	0.9300	C710—H71B	0.9600
C25—C26	1.378 (6)	C710—H71C	0.9600
C25—H25	0.9300	N81—C81	1.342 (6)
C26—C27	1.384 (6)	N81—C89	1.346 (6)
C26—H26	0.9300	C81—C82	1.397 (6)
C27—H27	0.9300	C81—H81	0.9300
O31—C31	1.265 (5)	C82—C83	1.339 (8)
O32—C31	1.265 (5)	C82—C810	1.505 (8)
C31—C32	1.491 (6)	C83—C84	1.416 (7)
C32—C33	1.376 (6)	C83—H83	0.9300
C32—C37	1.387 (6)	C84—C85	1.379 (8)
C33—C34	1.386 (6)	C84—C89	1.432 (6)
C33—H33	0.9300	C85—C86	1.387 (8)
C34—C35	1.381 (7)	C85—H85	0.9300
C34—H34	0.9300	C86—C87	1.365 (8)
C35—C36	1.363 (7)	C86—H86	0.9300
C35—H35	0.9300	C87—C88	1.379 (7)
C36—C37	1.378 (6)	C87—H87	0.9300
C36—H36	0.9300	C88—C89	1.381 (7)
C37—H37	0.9300	C88—H88	0.9300
O41—C41	1.260 (5)	C810—H81A	0.9600
O42—C41	1.256 (5)	C810—H81B	0.9600
C41—C42	1.492 (6)	C810—H81C	0.9600
C42—C43	1.362 (6)	N91—C91	1.341 (5)
C42—C47	1.378 (5)	N91—C99	1.352 (5)
C43—C44	1.382 (6)	C91—C92	1.397 (6)
C43—H43	0.9300	C91—H91	0.9300
C44—C45	1.383 (7)	C92—C93	1.357 (7)
C44—H44	0.9300	C92—C910	1.491 (8)
C45—C46	1.356 (7)	C93—C94	1.390 (7)
C45—H45	0.9300	C93—H93	0.9300
C46—C47	1.388 (6)	C94—C95	1.391 (7)
C46—H46	0.9300	C94—C99	1.420 (6)
C47—H47	0.9300	C95—C96	1.369 (8)
O51—C51	1.255 (5)	C95—H95	0.9300
O52—C51^i^	1.262 (5)	C96—C97	1.383 (7)
C51—O52^i^	1.262 (5)	C96—H96	0.9300
C51—C52	1.502 (6)	C97—C98	1.394 (7)
C52—C57	1.356 (6)	C97—H97	0.9300
C52—C53	1.373 (6)	C98—C99	1.377 (6)
C53—C54	1.393 (6)	C98—H98	0.9300
C53—H53	0.9300	C910—H91A	0.9600
C54—C55	1.365 (7)	C910—H91B	0.9600
C54—H54	0.9300	C910—H91C	0.9600
			
O31—Cu1—O11	167.94 (12)	C52—C53—H53	119.9
O31—Cu1—O41	88.60 (12)	C54—C53—H53	119.9
O11—Cu1—O41	90.36 (12)	C55—C54—C53	119.5 (5)
O31—Cu1—O21	90.61 (13)	C55—C54—H54	120.3
O11—Cu1—O21	87.62 (12)	C53—C54—H54	120.3
O41—Cu1—O21	166.54 (12)	C56—C55—C54	120.5 (5)
O31—Cu1—N71	93.60 (12)	C56—C55—H55	119.8
O11—Cu1—N71	98.43 (13)	C54—C55—H55	119.8
O41—Cu1—N71	99.22 (13)	C55—C56—C57	119.9 (5)
O21—Cu1—N71	94.24 (12)	C55—C56—H56	120.1
O31—Cu1—Cu2	81.57 (9)	C57—C56—H56	120.1
O11—Cu1—Cu2	86.37 (9)	C52—C57—C56	120.5 (5)
O41—Cu1—Cu2	86.09 (9)	C52—C57—H57	119.8
O21—Cu1—Cu2	80.50 (8)	C56—C57—H57	119.8
N71—Cu1—Cu2	172.77 (10)	C61—O61—Cu3	125.7 (3)
O22—Cu2—O32	91.12 (13)	C61^i^—O62—Cu3	120.5 (3)
O22—Cu2—O42	167.88 (12)	O62^i^—C61—O61	125.5 (4)
O32—Cu2—O42	88.38 (13)	O62^i^—C61—C62	117.2 (4)
O22—Cu2—O12	87.20 (13)	O61—C61—C62	117.3 (4)
O32—Cu2—O12	166.44 (12)	C67—C62—C63	119.6 (4)
O42—Cu2—O12	90.45 (13)	C67—C62—C61	120.1 (4)
O22—Cu2—N81	102.32 (13)	C63—C62—C61	120.2 (4)
O32—Cu2—N81	100.15 (13)	C62—C63—C64	120.3 (5)
O42—Cu2—N81	89.69 (13)	C62—C63—H63	119.9
O12—Cu2—N81	93.36 (13)	C64—C63—H63	119.9
O22—Cu2—Cu1	86.93 (9)	C65—C64—C63	119.8 (5)
O32—Cu2—Cu1	85.69 (9)	C65—C64—H64	120.1
O42—Cu2—Cu1	80.95 (9)	C63—C64—H64	120.1
O12—Cu2—Cu1	80.78 (9)	C64—C65—C66	120.6 (5)
N81—Cu2—Cu1	168.86 (12)	C64—C65—H65	119.7
O51—Cu3—O62	90.97 (12)	C66—C65—H65	119.7
O51—Cu3—O52	167.23 (12)	C67—C66—C65	119.7 (5)
O62—Cu3—O52	87.43 (13)	C67—C66—H66	120.1
O51—Cu3—O61	88.38 (13)	C65—C66—H66	120.1
O62—Cu3—O61	167.38 (12)	C62—C67—C66	120.0 (5)
O52—Cu3—O61	90.43 (13)	C62—C67—H67	120.0
O51—Cu3—N91	98.19 (13)	C66—C67—H67	120.0
O62—Cu3—N91	99.82 (13)	C71—N71—C79	118.0 (4)
O52—Cu3—N91	94.57 (13)	C71—N71—Cu1	113.2 (3)
O61—Cu3—N91	92.75 (13)	C79—N71—Cu1	128.7 (3)
O51—Cu3—Cu3^i^	85.14 (9)	N71—C71—C72	124.3 (5)
O62—Cu3—Cu3^i^	86.44 (9)	N71—C71—H71	117.9
O52—Cu3—Cu3^i^	82.12 (9)	C72—C71—H71	117.9
O61—Cu3—Cu3^i^	80.95 (9)	C73—C72—C71	117.6 (5)
N91—Cu3—Cu3^i^	172.82 (11)	C73—C72—C710	123.8 (5)
C11—O11—Cu1	121.0 (3)	C71—C72—C710	118.6 (5)
C11—O12—Cu2	126.6 (3)	C72—C73—C74	122.2 (5)
O12—C11—O11	124.4 (4)	C72—C73—H73	118.9
O12—C11—C12	118.4 (4)	C74—C73—H73	118.9
O11—C11—C12	117.2 (4)	C75—C74—C73	124.7 (5)
C17—C12—C13	119.0 (4)	C75—C74—C79	119.2 (5)
C17—C12—C11	119.1 (4)	C73—C74—C79	116.2 (5)
C13—C12—C11	121.8 (4)	C76—C75—C74	120.4 (5)
C14—C13—C12	120.2 (4)	C76—C75—H75	119.8
C14—C13—H13	119.9	C74—C75—H75	119.8
C12—C13—H13	119.9	C75—C76—C77	120.4 (6)
C15—C14—C13	120.6 (5)	C75—C76—H76	119.8
C15—C14—H14	119.7	C77—C76—H76	119.8
C13—C14—H14	119.7	C78—C77—C76	120.1 (5)
C16—C15—C14	119.3 (5)	C78—C77—H77	120.0
C16—C15—H15	120.4	C76—C77—H77	120.0
C14—C15—H15	120.4	C77—C78—C79	121.1 (5)
C15—C16—C17	120.5 (5)	C77—C78—H78	119.4
C15—C16—H16	119.7	C79—C78—H78	119.4
C17—C16—H16	119.7	N71—C79—C78	119.5 (4)
C12—C17—C16	120.4 (5)	N71—C79—C74	121.6 (4)
C12—C17—H17	119.8	C78—C79—C74	118.8 (5)
C16—C17—H17	119.8	C72—C710—H71A	109.5
C21—O21—Cu1	125.6 (3)	C72—C710—H71B	109.5
C21—O22—Cu2	120.0 (3)	H71A—C710—H71B	109.5
O21—C21—O22	125.5 (4)	C72—C710—H71C	109.5
O21—C21—C22	118.0 (4)	H71A—C710—H71C	109.5
O22—C21—C22	116.5 (4)	H71B—C710—H71C	109.5
C27—C22—C23	118.3 (4)	C81—N81—C89	117.7 (4)
C27—C22—C21	120.5 (4)	C81—N81—Cu2	112.5 (3)
C23—C22—C21	121.1 (4)	C89—N81—Cu2	129.0 (3)
C24—C23—C22	120.8 (4)	N81—C81—C82	125.2 (6)
C24—C23—H23	119.6	N81—C81—H81	117.4
C22—C23—H23	119.6	C82—C81—H81	117.4
C25—C24—C23	120.2 (5)	C83—C82—C81	116.8 (6)
C25—C24—H24	119.9	C83—C82—C810	124.5 (6)
C23—C24—H24	119.9	C81—C82—C810	118.6 (6)
C24—C25—C26	120.4 (5)	C82—C83—C84	121.9 (5)
C24—C25—H25	119.8	C82—C83—H83	119.1
C26—C25—H25	119.8	C84—C83—H83	119.1
C25—C26—C27	119.4 (5)	C85—C84—C83	124.1 (6)
C25—C26—H26	120.3	C85—C84—C89	119.0 (6)
C27—C26—H26	120.3	C83—C84—C89	116.8 (5)
C22—C27—C26	120.9 (4)	C84—C85—C86	120.7 (6)
C22—C27—H27	119.6	C84—C85—H85	119.7
C26—C27—H27	119.6	C86—C85—H85	119.7
C31—O31—Cu1	126.6 (3)	C87—C86—C85	120.6 (7)
C31—O32—Cu2	120.9 (3)	C87—C86—H86	119.7
O31—C31—O32	124.7 (4)	C85—C86—H86	119.7
O31—C31—C32	117.0 (4)	C86—C87—C88	119.6 (7)
O32—C31—C32	118.3 (4)	C86—C87—H87	120.2
C33—C32—C37	119.1 (4)	C88—C87—H87	120.2
C33—C32—C31	119.7 (5)	C87—C88—C89	121.8 (5)
C37—C32—C31	121.2 (4)	C87—C88—H88	119.1
C32—C33—C34	120.2 (5)	C89—C88—H88	119.1
C32—C33—H33	119.9	N81—C89—C88	120.3 (5)
C34—C33—H33	119.9	N81—C89—C84	121.5 (5)
C35—C34—C33	119.9 (5)	C88—C89—C84	118.2 (5)
C35—C34—H34	120.0	C82—C810—H81A	109.5
C33—C34—H34	120.0	C82—C810—H81B	109.5
C36—C35—C34	120.0 (5)	H81A—C810—H81B	109.5
C36—C35—H35	120.0	C82—C810—H81C	109.5
C34—C35—H35	120.0	H81A—C810—H81C	109.5
C35—C36—C37	120.2 (6)	H81B—C810—H81C	109.5
C35—C36—H36	119.9	C91—N91—C99	117.9 (4)
C37—C36—H36	119.9	C91—N91—Cu3	112.0 (3)
C36—C37—C32	120.5 (5)	C99—N91—Cu3	129.6 (3)
C36—C37—H37	119.8	N91—C91—C92	124.8 (5)
C32—C37—H37	119.8	N91—C91—H91	117.6
C41—O41—Cu1	120.8 (3)	C92—C91—H91	117.6
C41—O42—Cu2	126.8 (3)	C93—C92—C91	116.8 (5)
O42—C41—O41	125.0 (4)	C93—C92—C910	124.8 (6)
O42—C41—C42	117.3 (4)	C91—C92—C910	118.4 (6)
O41—C41—C42	117.6 (4)	C92—C93—C94	121.3 (5)
C43—C42—C47	119.0 (4)	C92—C93—H93	119.4
C43—C42—C41	121.0 (4)	C94—C93—H93	119.4
C47—C42—C41	120.1 (4)	C93—C94—C95	124.1 (5)
C42—C43—C44	121.3 (5)	C93—C94—C99	118.4 (5)
C42—C43—H43	119.3	C95—C94—C99	117.5 (5)
C44—C43—H43	119.3	C96—C95—C94	122.5 (6)
C43—C44—C45	119.2 (5)	C96—C95—H95	118.8
C43—C44—H44	120.4	C94—C95—H95	118.8
C45—C44—H44	120.4	C95—C96—C97	119.6 (6)
C46—C45—C44	120.1 (5)	C95—C96—H96	120.2
C46—C45—H45	120.0	C97—C96—H96	120.2
C44—C45—H45	120.0	C96—C97—C98	119.7 (6)
C45—C46—C47	120.2 (5)	C96—C97—H97	120.1
C45—C46—H46	119.9	C98—C97—H97	120.1
C47—C46—H46	119.9	C99—C98—C97	120.7 (5)
C42—C47—C46	120.2 (5)	C99—C98—H98	119.7
C42—C47—H47	119.9	C97—C98—H98	119.7
C46—C47—H47	119.9	N91—C99—C98	119.2 (5)
C51—O51—Cu3	122.0 (3)	N91—C99—C94	120.9 (5)
C51^i^—O52—Cu3	125.3 (3)	C98—C99—C94	120.0 (5)
O51—C51—O52^i^	125.0 (4)	C92—C910—H91A	109.5
O51—C51—C52	118.0 (4)	C92—C910—H91B	109.5
O52^i^—C51—C52	117.0 (4)	H91A—C910—H91B	109.5
C57—C52—C53	119.4 (4)	C92—C910—H91C	109.5
C57—C52—C51	119.6 (4)	H91A—C910—H91C	109.5
C53—C52—C51	121.0 (4)	H91B—C910—H91C	109.5
C52—C53—C54	120.2 (5)		
			
O31—Cu1—Cu2—O22	95.60 (13)	Cu3—O51—C51—O52^i^	8.5 (6)
O11—Cu1—Cu2—O22	−84.64 (12)	Cu3—O51—C51—C52	−172.3 (3)
O41—Cu1—Cu2—O22	−175.24 (12)	O51—C51—C52—C57	160.5 (4)
O21—Cu1—Cu2—O22	3.55 (13)	O52^i^—C51—C52—C57	−20.2 (6)
O31—Cu1—Cu2—O32	4.25 (12)	O51—C51—C52—C53	−18.8 (6)
O11—Cu1—Cu2—O32	−175.99 (12)	O52^i^—C51—C52—C53	160.4 (4)
O41—Cu1—Cu2—O32	93.40 (12)	C57—C52—C53—C54	1.4 (8)
O21—Cu1—Cu2—O32	−87.80 (12)	C51—C52—C53—C54	−179.3 (4)
O31—Cu1—Cu2—O42	−84.79 (13)	C52—C53—C54—C55	−0.7 (8)
O11—Cu1—Cu2—O42	94.97 (13)	C53—C54—C55—C56	−1.4 (9)
O41—Cu1—Cu2—O42	4.36 (13)	C54—C55—C56—C57	2.8 (9)
N71—Cu1—Cu2—O42	−133.2 (7)	C53—C52—C57—C56	0.0 (7)
O31—Cu1—Cu2—O12	−176.74 (13)	C51—C52—C57—C56	−179.3 (4)
O11—Cu1—Cu2—O12	3.02 (12)	C55—C56—C57—C52	−2.1 (8)
O41—Cu1—Cu2—O12	−87.58 (13)	O51—Cu3—O61—C61	−79.6 (4)
O21—Cu1—Cu2—O12	91.21 (13)	O62—Cu3—O61—C61	7.6 (8)
O31—Cu1—Cu2—N81	−117.9 (5)	O52—Cu3—O61—C61	87.7 (4)
O11—Cu1—Cu2—N81	61.9 (5)	N91—Cu3—O61—C61	−177.7 (4)
O41—Cu1—Cu2—N81	−28.7 (5)	Cu3^i^—Cu3—O61—C61	5.7 (3)
O21—Cu1—Cu2—N81	150.1 (5)	O51—Cu3—O62—C61^i^	89.5 (3)
O31—Cu1—O11—C11	1.9 (8)	O52—Cu3—O62—C61^i^	−77.8 (3)
O41—Cu1—O11—C11	86.9 (3)	O61—Cu3—O62—C61^i^	2.6 (8)
O21—Cu1—O11—C11	−79.8 (3)	N91—Cu3—O62—C61^i^	−172.0 (3)
N71—Cu1—O11—C11	−173.8 (3)	Cu3^i^—Cu3—O62—C61^i^	4.5 (3)
Cu2—Cu1—O11—C11	0.8 (3)	Cu3—O61—C61—O62^i^	−11.7 (7)
O22—Cu2—O12—C11	78.8 (4)	Cu3—O61—C61—C62	167.5 (3)
O32—Cu2—O12—C11	−4.3 (8)	O62^i^—C61—C62—C67	13.7 (6)
O42—Cu2—O12—C11	−89.2 (4)	O61—C61—C62—C67	−165.5 (4)
N81—Cu2—O12—C11	−179.0 (4)	O62^i^—C61—C62—C63	−170.3 (4)
Cu1—Cu2—O12—C11	−8.5 (3)	O61—C61—C62—C63	10.5 (6)
Cu2—O12—C11—O11	12.3 (6)	C67—C62—C63—C64	−0.3 (7)
Cu2—O12—C11—C12	−168.7 (3)	C61—C62—C63—C64	−176.4 (4)
Cu1—O11—C11—O12	−7.4 (6)	C62—C63—C64—C65	1.3 (7)
Cu1—O11—C11—C12	173.6 (3)	C63—C64—C65—C66	−1.1 (8)
O12—C11—C12—C17	16.7 (6)	C64—C65—C66—C67	−0.1 (8)
O11—C11—C12—C17	−164.2 (4)	C63—C62—C67—C66	−0.8 (7)
O12—C11—C12—C13	−164.0 (4)	C61—C62—C67—C66	175.2 (4)
O11—C11—C12—C13	15.0 (6)	C65—C66—C67—C62	1.0 (8)
C17—C12—C13—C14	−2.2 (7)	O31—Cu1—N71—C71	−50.7 (3)
C11—C12—C13—C14	178.5 (4)	O11—Cu1—N71—C71	128.4 (3)
C12—C13—C14—C15	2.5 (7)	O41—Cu1—N71—C71	−139.8 (3)
C13—C14—C15—C16	−0.5 (8)	O21—Cu1—N71—C71	40.2 (3)
C14—C15—C16—C17	−1.7 (8)	O31—Cu1—N71—C79	133.0 (3)
C13—C12—C17—C16	0.0 (7)	O11—Cu1—N71—C79	−47.9 (3)
C11—C12—C17—C16	179.3 (4)	O41—Cu1—N71—C79	43.9 (3)
C15—C16—C17—C12	2.0 (8)	O21—Cu1—N71—C79	−136.1 (3)
O31—Cu1—O21—C21	−92.3 (4)	C79—N71—C71—C72	0.2 (7)
O11—Cu1—O21—C21	75.8 (4)	Cu1—N71—C71—C72	−176.6 (4)
O41—Cu1—O21—C21	−5.7 (8)	N71—C71—C72—C73	−1.8 (8)
N71—Cu1—O21—C21	174.1 (4)	N71—C71—C72—C710	178.4 (5)
Cu2—Cu1—O21—C21	−10.9 (3)	C71—C72—C73—C74	2.2 (8)
O32—Cu2—O22—C21	87.5 (3)	C710—C72—C73—C74	−178.0 (5)
O42—Cu2—O22—C21	0.1 (8)	C72—C73—C74—C75	178.6 (5)
O12—Cu2—O22—C21	−79.0 (3)	C72—C73—C74—C79	−1.0 (7)
N81—Cu2—O22—C21	−171.8 (3)	C73—C74—C75—C76	−177.8 (5)
Cu1—Cu2—O22—C21	1.9 (3)	C79—C74—C75—C76	1.8 (7)
Cu1—O21—C21—O22	16.9 (7)	C74—C75—C76—C77	−1.7 (8)
Cu1—O21—C21—C22	−162.1 (3)	C75—C76—C77—C78	0.4 (8)
Cu2—O22—C21—O21	−11.1 (6)	C76—C77—C78—C79	0.8 (7)
Cu2—O22—C21—C22	167.9 (3)	C71—N71—C79—C78	−178.6 (4)
O21—C21—C22—C27	167.2 (4)	Cu1—N71—C79—C78	−2.5 (6)
O22—C21—C22—C27	−11.9 (6)	C71—N71—C79—C74	1.1 (6)
O21—C21—C22—C23	−9.2 (6)	Cu1—N71—C79—C74	177.2 (3)
O22—C21—C22—C23	171.7 (4)	C77—C78—C79—N71	179.1 (4)
C27—C22—C23—C24	−0.3 (7)	C77—C78—C79—C74	−0.6 (6)
C21—C22—C23—C24	176.2 (4)	C75—C74—C79—N71	179.7 (4)
C22—C23—C24—C25	−0.3 (7)	C73—C74—C79—N71	−0.7 (6)
C23—C24—C25—C26	0.5 (8)	C75—C74—C79—C78	−0.6 (6)
C24—C25—C26—C27	−0.1 (8)	C73—C74—C79—C78	179.0 (4)
C23—C22—C27—C26	0.6 (7)	O22—Cu2—N81—C81	143.3 (3)
C21—C22—C27—C26	−175.8 (4)	O32—Cu2—N81—C81	−123.3 (3)
C25—C26—C27—C22	−0.5 (8)	O42—Cu2—N81—C81	−35.1 (3)
O11—Cu1—O31—C31	−4.4 (8)	O12—Cu2—N81—C81	55.4 (3)
O41—Cu1—O31—C31	−89.5 (3)	Cu1—Cu2—N81—C81	−2.4 (7)
O21—Cu1—O31—C31	77.1 (3)	O22—Cu2—N81—C89	−47.9 (4)
N71—Cu1—O31—C31	171.4 (3)	O32—Cu2—N81—C89	45.5 (4)
Cu2—Cu1—O31—C31	−3.2 (3)	O42—Cu2—N81—C89	133.8 (4)
O22—Cu2—O32—C31	−93.7 (3)	O12—Cu2—N81—C89	−135.8 (4)
O42—Cu2—O32—C31	74.2 (3)	Cu1—Cu2—N81—C89	166.4 (3)
O12—Cu2—O32—C31	−11.0 (7)	C89—N81—C81—C82	−1.6 (7)
N81—Cu2—O32—C31	163.5 (3)	Cu2—N81—C81—C82	168.6 (4)
Cu1—Cu2—O32—C31	−6.9 (3)	N81—C81—C82—C83	2.4 (8)
Cu1—O31—C31—O32	−1.1 (6)	N81—C81—C82—C810	−177.3 (5)
Cu1—O31—C31—C32	177.5 (3)	C81—C82—C83—C84	−0.8 (9)
Cu2—O32—C31—O31	7.0 (6)	C810—C82—C83—C84	178.8 (6)
Cu2—O32—C31—C32	−171.6 (3)	C82—C83—C84—C85	−179.0 (5)
O31—C31—C32—C33	−17.5 (6)	C82—C83—C84—C89	−1.2 (8)
O32—C31—C32—C33	161.2 (4)	C83—C84—C85—C86	178.7 (6)
O31—C31—C32—C37	162.2 (4)	C89—C84—C85—C86	1.0 (8)
O32—C31—C32—C37	−19.2 (6)	C84—C85—C86—C87	0.2 (10)
C37—C32—C33—C34	0.6 (7)	C85—C86—C87—C88	−1.1 (9)
C31—C32—C33—C34	−179.8 (4)	C86—C87—C88—C89	0.7 (8)
C32—C33—C34—C35	−2.5 (7)	C81—N81—C89—C88	−179.4 (4)
C33—C34—C35—C36	2.6 (8)	Cu2—N81—C89—C88	12.2 (6)
C34—C35—C36—C37	−0.8 (8)	C81—N81—C89—C84	−0.7 (6)
C35—C36—C37—C32	−1.1 (8)	Cu2—N81—C89—C84	−169.0 (3)
C33—C32—C37—C36	1.2 (7)	C87—C88—C89—N81	179.3 (4)
C31—C32—C37—C36	−178.4 (4)	C87—C88—C89—C84	0.5 (7)
O31—Cu1—O41—C41	76.4 (3)	C85—C84—C89—N81	179.9 (4)
O11—Cu1—O41—C41	−91.6 (3)	C83—C84—C89—N81	2.0 (7)
O21—Cu1—O41—C41	−10.4 (7)	C85—C84—C89—C88	−1.3 (7)
N71—Cu1—O41—C41	169.8 (3)	C83—C84—C89—C88	−179.2 (4)
Cu2—Cu1—O41—C41	−5.3 (3)	O51—Cu3—N91—C91	−126.5 (3)
O22—Cu2—O42—C41	−3.4 (8)	O62—Cu3—N91—C91	141.1 (3)
O32—Cu2—O42—C41	−91.2 (4)	O52—Cu3—N91—C91	52.9 (3)
O12—Cu2—O42—C41	75.3 (4)	O61—Cu3—N91—C91	−37.8 (3)
N81—Cu2—O42—C41	168.7 (4)	O51—Cu3—N91—C99	44.8 (4)
Cu1—Cu2—O42—C41	−5.3 (3)	O62—Cu3—N91—C99	−47.6 (4)
Cu2—O42—C41—O41	3.0 (7)	O52—Cu3—N91—C99	−135.8 (4)
Cu2—O42—C41—C42	−179.0 (3)	O61—Cu3—N91—C99	133.6 (4)
Cu1—O41—C41—O42	3.2 (6)	C99—N91—C91—C92	−0.6 (7)
Cu1—O41—C41—C42	−174.8 (3)	Cu3—N91—C91—C92	171.9 (4)
O42—C41—C42—C43	−175.4 (4)	N91—C91—C92—C93	−0.1 (8)
O41—C41—C42—C43	2.7 (6)	N91—C91—C92—C910	−178.8 (5)
O42—C41—C42—C47	4.8 (6)	C91—C92—C93—C94	1.0 (8)
O41—C41—C42—C47	−177.1 (4)	C910—C92—C93—C94	179.6 (6)
C47—C42—C43—C44	−0.1 (7)	C92—C93—C94—C95	179.4 (5)
C41—C42—C43—C44	−179.9 (4)	C92—C93—C94—C99	−1.2 (8)
C42—C43—C44—C45	−1.2 (8)	C93—C94—C95—C96	−180.0 (5)
C43—C44—C45—C46	1.1 (9)	C99—C94—C95—C96	0.7 (8)
C44—C45—C46—C47	0.3 (9)	C94—C95—C96—C97	−0.9 (9)
C43—C42—C47—C46	1.4 (7)	C95—C96—C97—C98	0.5 (8)
C41—C42—C47—C46	−178.7 (4)	C96—C97—C98—C99	0.1 (8)
C45—C46—C47—C42	−1.5 (8)	C91—N91—C99—C98	−179.7 (4)
O62—Cu3—O51—C51	−91.0 (3)	Cu3—N91—C99—C98	9.4 (6)
O52—Cu3—O51—C51	−8.4 (8)	C91—N91—C99—C94	0.3 (6)
O61—Cu3—O51—C51	76.4 (3)	Cu3—N91—C99—C94	−170.6 (3)
N91—Cu3—O51—C51	168.9 (3)	C97—C98—C99—N91	179.7 (4)
Cu3^i^—Cu3—O51—C51	−4.7 (3)	C97—C98—C99—C94	−0.3 (7)
O51—Cu3—O52—C51^i^	0.9 (8)	C93—C94—C99—N91	0.6 (7)
O62—Cu3—O52—C51^i^	84.0 (3)	C95—C94—C99—N91	179.9 (4)
O61—Cu3—O52—C51^i^	−83.6 (3)	C93—C94—C99—C98	−179.4 (4)
N91—Cu3—O52—C51^i^	−176.4 (3)	C95—C94—C99—C98	0.0 (6)
Cu3^i^—Cu3—O52—C51^i^	−2.8 (3)		

Symmetry codes: (i) −*x*+1, −*y*+1, −*z*+1.
